# Biological hallmarks of systemic sclerosis are present in the skin and serum of patients with Very Early Diagnosis of Systemic Sclerosis (VEDOSS)

**DOI:** 10.1093/rheumatology/keae698

**Published:** 2024-12-19

**Authors:** Rebecca L Ross, Begoña Caballero-Ruiz, Emily L Clarke, Vishal Kakkar, Christopher W Wasson, Panji Mulipa, Enrico De Lorenzis, Will Merchant, Stefano Di Donato, Andrea Rindone, Ariane L Herrick, Christopher P Denton, Natalia A Riobo-Del Galdo, Francesco Del Galdo

**Affiliations:** Leeds Institute of Rheumatic and Musculoskeletal Medicine, University of Leeds, Leeds, UK; NIHR Leeds Biomedical Research Centre, Leeds Teaching Hospitals, NHS Trust, Chapel Allerton Hospital, Leeds, UK; Leeds Institute of Rheumatic and Musculoskeletal Medicine, University of Leeds, Leeds, UK; Section of Pathology and Tumour Biology, University of Leeds, Leeds, UK; Leeds Teaching Hospitals, NHS Trust, St James’s University Hospital, Leeds, UK; Leeds Institute of Rheumatic and Musculoskeletal Medicine, University of Leeds, Leeds, UK; Leeds Institute of Rheumatic and Musculoskeletal Medicine, University of Leeds, Leeds, UK; Leeds Institute of Rheumatic and Musculoskeletal Medicine, University of Leeds, Leeds, UK; Leeds Institute of Rheumatic and Musculoskeletal Medicine, University of Leeds, Leeds, UK; Division of Rheumatology, Catholic University of the Sacred Heart, Fondazione Policlinico Universitario A. Gemelli, IRCCS, Rome, Italy; Leeds Teaching Hospitals, NHS Trust, St James’s University Hospital, Leeds, UK; Leeds Institute of Rheumatic and Musculoskeletal Medicine, University of Leeds, Leeds, UK; Leeds Institute of Rheumatic and Musculoskeletal Medicine, University of Leeds, Leeds, UK; Department of Rheumatology and Medical Science, University of Milan, ASST Gaetano Pini-CTO Institute, Milan, Italy; Division of Musculoskeletal & Dermatological Sciences, The University of Manchester, Northern Care Alliance NHS Foundation Trust, Manchester Academic Health Science Centre, Manchester, UK; Centre for Rheumatology, Division of Medicine, University College London, London, UK; School of Molecular and Cellular Biology, Faculty of Biological Sciences, University of Leeds, Leeds, UK; Leeds Institute of Medical Research, University of Leeds, Leeds, UK; Astbury Centre for Structural Molecular Biology, University of Leeds, Leeds, UK; Leeds Institute of Rheumatic and Musculoskeletal Medicine, University of Leeds, Leeds, UK; NIHR Leeds Biomedical Research Centre, Leeds Teaching Hospitals, NHS Trust, Chapel Allerton Hospital, Leeds, UK

**Keywords:** systemic sclerosis, autoimmune diseases, fibrosis, connective tissue diseases, VEDOSS, interferon, CXCL10, extracellular matrix, collagen, dermal fibroblasts

## Abstract

**Objective:**

The Very Early Diagnosis of Systemic Sclerosis (VEDOSS) EUSTAR study showed that, despite not showing any clinical sign of disease, patients with Raynaud’s and ANA and/or capillaroscopy abnormalities often progress to SSc within 5 years. We aimed to determine whether VEDOSS biosamples show biological SSc activity pre-clinically.

**Methods:**

Skin biopsies were histologically analysed. Dermal fibroblasts analysed by RT-qPCR and gel contraction assays. Sera were assayed by Luminex (CXCL10) or ELISA (ELF score). Healthy controls (HC) and SSc biosamples were used for controls.

**Results:**

Overall, 114 consecutive VEDOSS patients were enrolled, of which 36 consented to have skin biopsies. Skin biopsies showed a variable but overall increased collagen staining and skin thickness, increased perivascular infiltrate of CD45-positive cells and CXCL10 expression. *In vitro*, VEDOSS dermal fibroblasts showed increased profibrotic gene expression and contractibility compared with HC. Increased serological CXCL10 [mean (s.d.) 75.90 (107.80) *vs* HC 39.90 (26.27) pg/ml, *P* = 0.02] and ELF score was evident in VEDOSS compared with HC [8.19 (0.78) *vs* 8.55 (0.79), *P* = 0.04]. In longitudinal analysis of a median of 27.5 (interquartile range 44.5) months, 14.9% of VEDOSS patients progressed to SSc. Baseline CXCL10 serum concentration was significantly higher in the VEDOSS patients that progressed (2-fold increase, *P* = 0.0071) and correlated with ELF score (R = 0.3096, *P* = 0.0065).

**Conclusions:**

Despite not fulfilling classification criteria, VEDOSS patients show SSc-linked fibrosis and immunity dysregulation both within the tissue and sera, supporting a biological diagnosis of disease and a window of opportunity to detect the biological pathways amenable for preventive intervention.

Rheumatology key messages

- Early-stage fibrosis and inflammation is evident in VEDOSS skin and sera.
- Type I IFN activation is frequent within the skin and sera of VEDOSS patients.
- Extracellular matrix remodelling and Type I IFN activation correlate in VEDOSS samples.

## Introduction

SSc is a highly variable autoimmune condition characterized by tissue and vascular fibrosis, carrying the highest morbidity and mortality among rheumatic diseases [1, 2]. The diagnosis of SSc relies on the identification of clinical signs of tissue and vascular fibrosis, including detection of skin thickness through the modified Rodnan skin score (mRSS), interstitial lung disease through high resolution CT scanning and vascular manifestations including detection of digital ulcers or increased pulmonary artery pressure. The detection of clinical signs of tissue and vascular fibrosis is not a direct sign of the autoimmune process driving their onset, and as such it happens inevitably late in the pathogenesis of the disease. The irreversible nature of most fibrotic manifestations and the lateness in establishing a therapeutic intervention may contribute to the limited effectiveness of disease-modifying approaches, especially for treatment targeting the immune or inflammatory process [3]. Indeed, indirect evidence from recent trials of tocilizumab in SSc-interstitial lung disease (ILD) does support the notion that clinical outcome may improve with an earlier therapeutic intervention, prior to irreversible organ damage [4–6]. With the aim of supporting earlier therapeutic intervention, in 2013 a EULAR and ACR taskforce endorsed a substantial revision of the classification criteria originally published in 1980, resulting in an increased sensitivity for an earlier classification of SSc [5–9].

RP occurs in >90% of SSc, most frequently preceding the clinical manifestations of tissue and vascular fibrosis by several years [10–12]. The EUSTAR multicentre Very Early Diagnosis of Systemic Sclerosis (VEDOSS) study indicated strong evidence that the presence of ANA in patients with RP was associated with 59% risk of fulfilling classification criteria within 5 years *vs* 11% of patients without ANA. This proportion increased to over 70% if patients showed either SSc-specific ANAs [SSc-AB, such as ACA, anti–topoisomerase I (anti-Scl70) and anti–RNA polymerase III (anti-RNAPOL III)], puffy fingers (PF) or abnormal nailfold videocapillaroscopy (NVC) findings [9, 12, 13]. The risk was proportionally higher if any of these features were present in combination with the others, up to 94% progression in RP patients with SSc-Ab and PF at baseline [14]. The results of the VEDOSS study effectively defined a population at risk of developing the clinical complications associated with SSc and informed the opportunity to design interception studies to prevent clinical manifestations of SSc. Nevertheless, there is limited information on the biological activity of SSc in this patient population. Previous studies have shown that the serum concentration of CXCL10 is increased in VEDOSS (*n* = 21) [15] and is increased up to 5 years before SSc clinical diagnosis [16]. More recently, serological markers of extracellular matrix (ECM) remodelling were shown to be dysregulated in VEDOSS (*n* = 42 cohort), but not shown to be predictive of progression [17].

Here we aimed to study biosamples from VEDOSS patients to determine whether the pathognomonic pathological signs of SSc can be detected within the skin, dermal fibroblasts and sera from VEDOSS patients and inform the rationale for multiomic approaches to identify the active biological pathways amenable for preventive intervention.

## Methods

### Patient enrolment and clinical characterization

Study patients (*n* = 114) were consecutively enrolled from a national VEDOSS inception cohort within the observational study STRIKE (Kennedy Cohort for Prevention of Systemic Sclerosis) through three UK-based centres (Leeds, Manchester and London) [18]. All participants provided written informed consent according to a protocol approved by Medicine and Health Regulatory agency (NRES-011NE to FDG, IRAS 15/NE/0211). Patients were included in the at-risk population if they presented with Raynaud’s and any VEDOSS criteria [9, 12, 13], while still not meeting 2013 ACR/EULAR classification criteria for SSc (score <9); they had mRSS = 0 and did not fulfil classification for any other CTD. Clinical data were collected according to the EUSTAR MEDS [19] deidentified and stored in an electronic database (Macro, Elsevier). Consented patients were approached to donate serum samples and an optional skin biopsy. All patients underwent eight fingers NVC imaging which was scored for the presence of an SSc pattern according to Cutolo *et al.* [20].

### Skin biopsies

Up to two full thickness skin biopsies were taken from the forearm dorsal skin using a 3-mm punch for each patient who consented to the procedure (*n* = 36). One biopsy was used for histological assessment while the other was employed for fibroblast isolation. Skin biopsies from healthy controls (HC) or patients fulfilling SSc criteria were employed as controls. Fibroblasts were isolated from skin biopsies (*n* = 6) by expansion out of scalpel-cut biopsies, and primary cell lines established after two passages. Isolated fibroblasts were maintained in DMEM with 10% fetal bovine serum and 1% penicillin/streptomycin and passaged at 80% confluency. All experiments on primary dermal fibroblasts were performed within five passages. Human telomerase-immortalization was carried out to immortalize SSc dermal fibroblasts using retroviral transduction as described [21].

### Histology

Biopsies were formalin-fixed and embedded in paraffin. Sequential sections were cut at 5 μm. Sequential sections were subjected to haematoxylin and eosin (H&E) staining (VEDOSS *n* = 36: HC *n* = 20, SSc *n* = 6), Masson’s trichrome (MT) to stain collagen blue and muscle red to identify the extent of fibrosis in the skin samples (VEDOSS *n* = 36: HC *n* = 5, SSc *n* = 6), and two others for immunohistochemistry (IHC). IHC involved antigen retrieval using sodium citrate. Sections were stained with CD45 (VEDOSS *n* = 10: HC *n* = 4, SSc *n* = 6), and CXCL10 (VEDOSS *n* = 10: HC *n* = 3) (Abcam) antibodies followed by ImmPRESS™ (Peroxidase) Polymer Anti-Rabbit IgG Reagent (Vector Laboratories), and visualized with 3, 3′-diaminobenzidine (DAB) (Vector Laboratories). Slides were scanned using a Leica Biosystems (Wetzlar, Germany) AT2 digital slide scanner at ×20 resolution.

### Quantification of histology

H&E-stained slides were reviewed on a Jusha (Nanjing, China) 31″ medical grade display by an expert dermatopathologist blinded to clinical data, to assess for features of SSc. A blinded probabilistic image analysis model was used to detect areas of brown immunopositivity (CD45 immunostaining) and blue staining collagen (MT stain) within the samples, using HeteroGenius Medical Imaging Manager (MIM) colour analysis add-on (HeteroGenius, Leeds, UK). Sequential manual annotation was used to train the algorithm until the performance was optimized. The dermal area was manually annotated, excluding epidermis, fat and areas of haemorrhage. The model was applied within the annotated dermal area to determine the area of blue staining collagen or immunopositivity in square microns of dermis area. Skin thickness was measured using H&E-stained section. Skin thickness was quantified through ImageJ, using 10 vertical measurements (µm) equally spread through the cross section of the skin, from the basement membrane to fatty structures. Mean thickness values were combined for each patient subset for group statistics. Scoring was done independently of MT staining. Dermal CXCL10 semiquantitative analysis was performed by an independent analyst on areas excluding fat tissue and glands. Staining was quantified using Fiji software (ImageJ2, 2.14.0/1.54F) using the colour deconvolution and analyse particles tools. The analysed particles were measured as a percentage of total area within two regions for each sample, which were used to derive the mean per sample.

### CXCL10 sera quantification

Sera samples from VEDOSS patients (*n* = 114) were assayed using a Human Magnetic Luminex xMAP assay to measure the concentration of CXCL10 (Bio-techne, Oxford, UK), according to manufacturer’s instructions and analysed using a Luminex 200 instrument with xPonent 4.2. Sera from HC (*n* = 93) and SSc (*n* = 284) patients were employed as controls.

### ELF score

ELF score was produced by the measurement of the sera levels of tissue inhibitor of metalloproteinases 1 (TIMP-1), amino-terminal propeptide of type III procollagen (PIIINP) and hyaluronic acid (HA), through automated high throughput diagnostics (Siemens Alpha-Centaur). ELF score was conducted on a smaller cohort of patient samples within those with CXCL10 serological analysis (VEDOSS, HC and SSc; *n* = 77, 22 and 143, respectively).

### Quantitative real time PCR

RNA was extracted from cells using the RNA extraction kit (Zymo Research) following the manufacturing protocols. RNA was reverse transcribed using the cDNA synthesis kit with hexarandom primers (Thermo). RT-qPCR was performed using SyBr Green PCR kit (Thermo) with primers specific for *COL1A1* (forward, CCTCCAGGGCTCCAACGAG; reverse, TCTATCACTGTCTTGCCCCA), *COL1A2* (forward, GATGTTGAACTTGTTGCTGAGC; reverse, TCTTTCCCCATTCATTTGTCTT), *ACTA2* (forward, TGTATGTGGCTATCCAGGCG; reverse, AGAGTCCAGCACGATGCCAG), *CCN2* (forward, GTGTGCACTGCCAAAGATGGT; reverse, TTGGAAGGACTCACCGCT) and *GAPDH* (forward, ACCCACTCCTCCACCTTTGA; reverse, CTGTTGCTGTAGCCAAATTCGT). The data obtained were analysed according to the ΔΔCt method. *GAPDH* served as housekeeping gene.

### Collagen gel contraction assay

Collagen gel contraction assays were prepared using Cell contraction Assay Kits (Cell Biolabs), per manufacturer instructions. Briefly, 2 × 10^5^ fibroblasts were cultured within collagen gel for 16 h at 37°C 5% CO_2_, then released from the sides of wells and photos taken over 72 h. The percentage change in gel area relative to area of gel at 0 h was analysed with ImageJ software.

### Statistical analysis

Categorical variables were presented as numbers and percentages, while continuous variables were reported as mean ± s.d., mean ± s.e. or median with interquartile range (IQR) depending on the data distribution. Comparisons between two groups, Student’s *t-*test was used. For comparisons between more than two groups, one-way ANOVA was used. The relationship between continuous variables was explored using Pearson and Spearman correlation coefficients. Statistical significance was defined as a *P*-value <0.05 for all analyses, and all tests were two-tailed. Data analysis was performed using RStudio (version 2023.03.0) or GraphPad Prism software (version 9.5.1).

## Results

### Patient population

VEDOSS patients (*n* = 114) were enrolled between January 2016 and June 2023. All patients consented to serum sampling and 36 patients consented to skin biopsy collection and analysis. The demographic and clinical features of VEDOSS patients are summarized in Table 1. Baseline clinical data was collected, including presence of anti-nuclear antibodies, NVC patterns, presence of PF and telangiectasias, and lung function tests [forced vital capacity (FVC) and alveolar diffusion of carbon monoxide (DLCO)]. Longitudinal analysis on at least one follow-up visit was performed on all 114 VEDOSS patients, with a median follow-up duration of 27.5 months (ranging 2.4–93.6 months). Clinical analysis for disease progression was last performed in November 2023 before reporting these results, within which 16/114 patients (14%) progressed to fulfil classification criteria for SSc, within a median 10.5 months (IQR 21 months).

**Table 1.** VEDOSS patient cohort clinical characteristics

Clinical characteristics	VEDOSS serum (*n* = 114)	VEDOSS biopsies (*n* = 36)
Age	48.7 ± 11.7	46.8 ± 11.9
Female (%)	89.5	91.7
ANA (%)	95.6	100
ACA	63.3	55.6
Scl70	22.0	38.9
Abnormal NVC (%)	37.2	34.3
Early NVC pattern	65.1	66.7
Active NVC pattern	34.9	33.3
Puffy fingers (%)	8.1	18.2
Telangiectasias (%)	4.5	6.1
FVC%	111 ± 17.7	109.0 ± 19.9
DLCO%	81.4 ± 15.1	87.6 ± 15.6
ACR-EULAR 2013 score	6.6 ± 1.3	6.8 ± 1.0
Follow-up period	27.5 ± 26.4	15.5 ± 20.5
Proportion of progressors (%)	14	5.6
Median time of progression	10.5 ± 21.3	8.5 ± 9.2

Age, FVC% and DLCO% and A-E 2013 SCORE expressed as mean ± s.d. All other characteristics shown as percentage of VEDOSS samples within group. ACA and Scl70 percentages are calculated from those with ANA positivity. Early and active NVC pattern percentages are calculated from those with abnormal NVC. Missing baseline clinical data for 1 patient for NVC analysis, 3 patients for puffy fingers and telangiectasias, and 23 patients for lung analysis. Follow-up period and median time of progression is from date of sample collection (at enrolment) to analysis of follow-up clinical features, shown in months±s.d. Progression determined at subsequent follow-up clinics and determined if A-E 2013 score increases to 9 and above. VEDOSS: Very Early Diagnosis of Systemic Sclerosis; Scl70: anti-topoisomerase; NVC: nailfold videocapillaroscopy; FVC: forced vital capacity; DLCO: alveolar diffusion of carbon monoxide.

### Biopsies from VEDOSS patients show increased extracellular matrix deposition and thickness compared with HC

**Figure 1.**
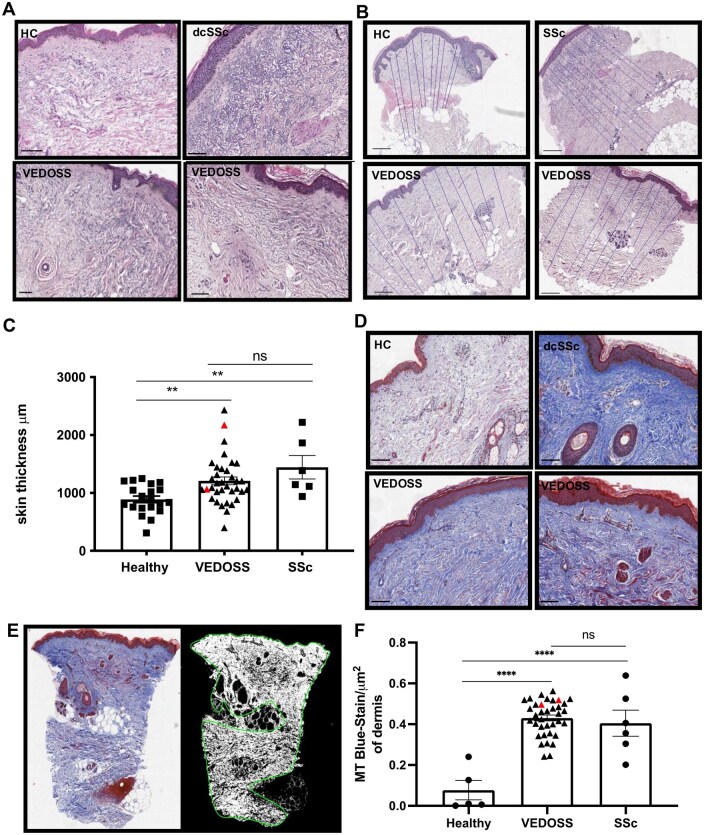
Skin biopsies from VEDOSS patients have increased dermal collagen and skin thickness compared with HC, similar to that seen in SSc. Three-millimetre skin punch biopsies were taken from the forearms of 36 VEDOSS patients, 20 HC and 6 SSc patients (dcSSc 5 and lcSSc 1). VEDOSS demographic and clinical classifications depicted in Table 1. (**A**) Representative H&E staining. Scale bar depicts 100 µm. See Supplementary Fig. S1A, available at *Rheumatology* online for HC *n* = 5, VEDOSS *n* = 10, SSc *n* = 5 additional sample staining. (**B**) H&E representative images with measurement of skin thickness (basement membrane to the fat layers); 10 measurements per biopsy. Scale bar depicts 200 µm. (**C**) Skin thickness analysis of all suitable samples within the three categories with population (HC *n* = 20, VEDOSS *n* = 35, SSc *n* = 6). See Supplementary Fig. S1B, available at *Rheumatology* online for example spread of skin thickness in representative samples (*n* = 9). (**D**) Representative MT staining. See Supplementary Fig. S2, available at *Rheumatology* online for additional sample staining. Scale bar depicts 100 µm. (**E**) Representative image showing a blinded and probabilistic image analysis model was used to detect areas of blue MT staining within a defined area, excluding fat and epidermis, using HeteroGenius Medical Imaging Manager colour analysis. (**F**) Quantification of collagen staining (as in E) per µm^2^ of dermis plotted (HC *n* = 5, VEDOSS *n* = 36, SSc *n* = 6). Graphs show mean ± s.e.m., single points represent individual biosamples, with red highlighting those baseline VEDOSS samples that progressed to SSc. One-way ANOVA used for analysis (**P < 0.01, ****P < 0.0001). VEDOSS: Very Early Diagnosis of Systemic Sclerosis; HC: healthy controls; H&E: haematoxylin and eosin; MT: Masson’s trichrome

Prior to clinical follow-up analysis for progression, available sectioned VEDOSS skin biopsies were assessed for biological hallmarks of SSc. H&E staining was performed on HC (*n* = 20), VEDOSS (*n* = 36) and SSc samples (5 dcSSc and one with lcSSc, all with local mRSS ≥1). Representative H&E images are shown in Fig. 1A and Supplementary Fig. S1, available at *Rheumatology* online. One VEDOSS sample did not meet full coverage of the skin architecture and was excluded from histology analysis. An expert dermatopathologist (W.M.), blinded to classification criteria assessed and scored H&E-stained sections for histopathological hallmarks supporting the diagnosis of SSc including dense collagen bundles arranged in parallel, dermal papillary flattening, loss of fat around eccrine coils, loss of adipose tissue and increased cellularity, perivascular inflammation and loss of a dermal ‘waist’ post-fixation [22–27]. Some 57% of VEDOSS samples showed more than one feature supporting a diagnosis of SSc, with 9% of VEDOSS samples displaying all the diagnostic features (Supplementary Fig. S1, available at *Rheumatology* online). For comparison, HC samples showed no SSc diagnosis (Supplementary Fig. S1, available at *Rheumatology* online).

Skin thickness was conducted on all biopsies showing full depth coverage (HC, VEDOSS, SSc; *n* = 20, 35, 6, respectively) (Fig. 1E). The skin thickness of VEDOSS samples was significantly greater than HC (mean ± SEM; 1.2 ± 0.1 mm *vs* 0.9 ± 0.1 mm, *P* = 0.0024), similar to that seen in SSc (1.4 ± 0.2 mm, *P* = 0.0019) (Fig. 1B and C). Thus, increased ECM deposition is already detectable within the dermis of VEDOSS patients, supporting fibrotic skin involvement despite no clinically detectable skin thickening.

MT staining was further conducted to specifically assess the density of ECM in the dermis of VEDOSS patients (*n* = 36) compared with representative SSc (*n* = 6) and HC (*n* = 5) controls (Fig. 1D and Supplementary Fig. S2, available at *Rheumatology* online). Visual and semiquantitative image analysis indicated that VEDOSS samples showed a mean 5.6-fold increased collagen dermal staining, compared with HC (*P* *<* 0.0001), similar to what is observed in SSc samples with local clinically detectable skin involvement (mRSS ≥1; 5.3-fold; *P* *<* 0.0001) (Fig. 1E and F).

### Explanted dermal fibroblasts from VEDOSS patients show profibrotic activation as observed in SSc dermal fibroblasts

**Figure 2.**
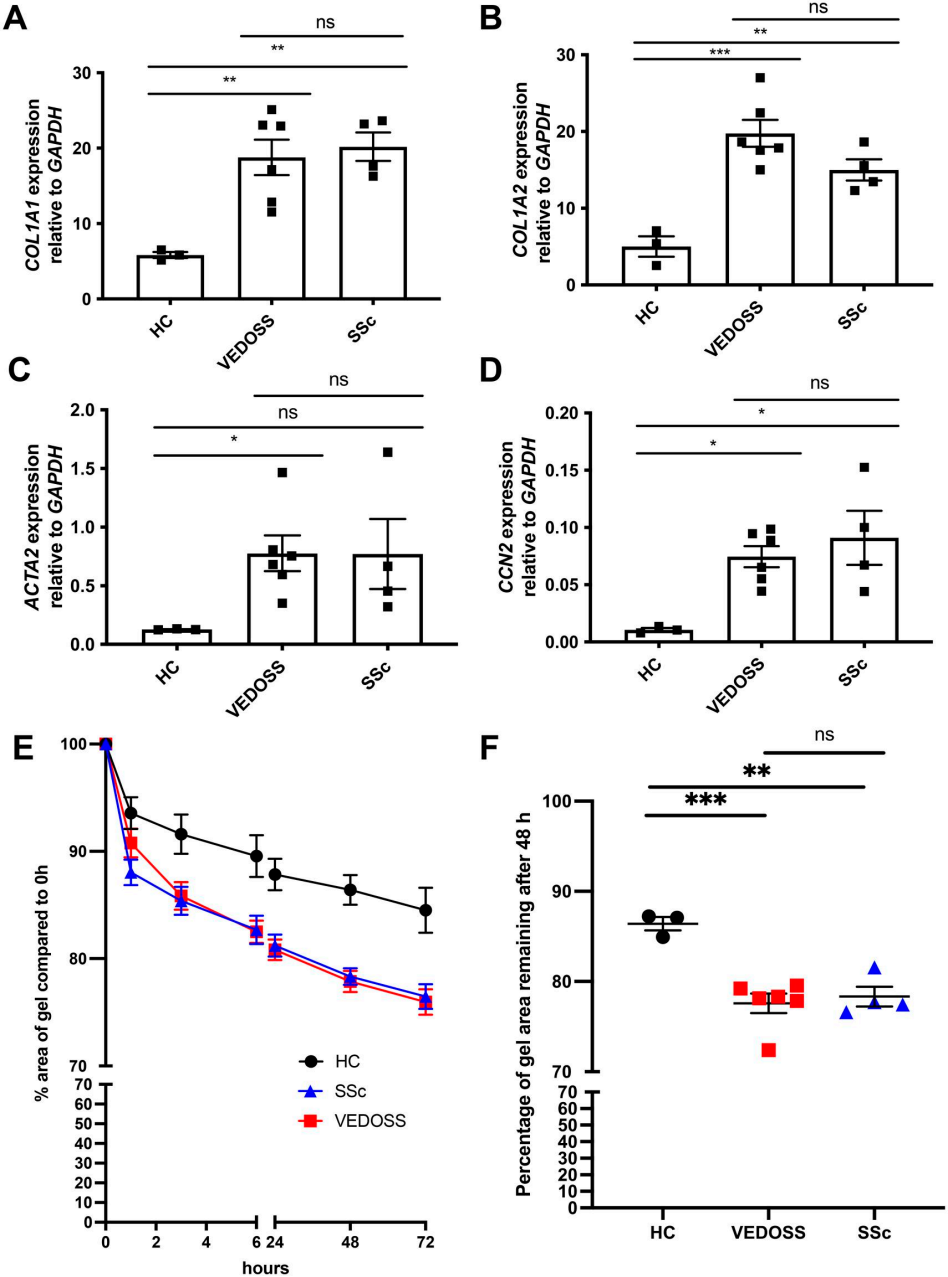
Explanted dermal fibroblasts from VEDOSS patients show increased profibrotic gene expression and increased contractility compared with HC. (**A**–**D**) *COL1A1*, *COL1A2*, *ACTA2* and *CCN2* mRNA expression in fibroblasts cultured in serum-starved media relative to *GAPDH* housekeeping gene. Data shows biological replicates of each subset (single dots), population mean ±  s.e.m. (**E**) Contractility of HC, VEDOSS and SSc fibroblasts, measured by percentage of gel area compared with 0 h, over 72 h. Each cell line (*n* = 3–6) repeated in triplicates. (**F**) Illustrates results of (C) at 48h. Data shows mean of subsets ± s.e.m., single dots represent the mean values for individual biosamples. One-way ANOVA used for analysis (ns = non-significant; *P < 0.05, **P < 0.01, ***P < 0.001). VEDOSS: Very Early Diagnosis of Systemic Sclerosis; HC: healthy controls; ANOVA: analysis of variance

Dermal fibroblasts explanted from SSc skin biopsies show a profibrotic activation *in vitro*, which has been extensively studied over the years [28]. The prototypical markers of this profibrotic activation include increased mRNA and protein expression of collagens type 1 (*COL1A1*, *COL1A2*), connective tissue growth factor (CTGF, encoded by *CCN2*) and α-SMA (encoded by *ACTA2*) expression [29–31]. Dermal fibroblasts explanted from skin biopsies of VEDOSS patients (*n* = 6), showed 3- to 7-fold increase in profibrotic gene expression (*COL1A1*, *COL1A2*, *ACTA2* and *CCN2*) relative to HC (Fig. 2A–D), similar to SSc fibroblasts (*N* = 4). Notably, human telomerase (HTERT)-mediated immortalization of these cells did not affect their profibrotic features (Supplementary Fig. S3, available at *Rheumatology* online). Functionally, we assessed the contractility of primary dermal fibroblasts using collagen gel matrices. Similar to what is already known in SSc, dermal fibroblasts from VEDOSS skin biopsies displayed a significantly stronger contraction of the collagen gel compared with HC (Fig. 2E and F, Supplementary Fig. S4, available at *Rheumatology* online).

### Skin biopsies from VEDOSS patients show increased inflammatory cell infiltrate linked with increased collagen and CXCL10 dermal expression

**Figure 3.**
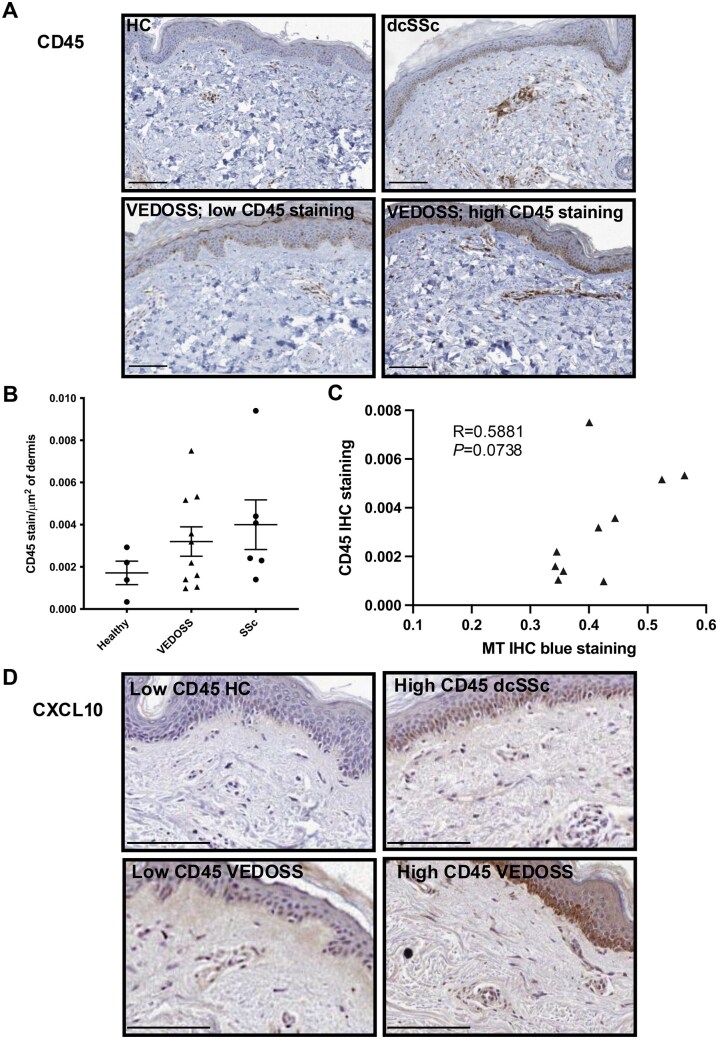
Skin biopsies of VEDOSS patients have increased perivascular infiltration, linked with increased collagen deposition and increased CXCL10 IFN-induced protein expression. Representative IHC staining for CD45 staining for each patient category, with VEDOSS showing representative images of low and high CD45 staining (**A**). See Supplementary Fig. S5A, available at *Rheumatology* online for all sample staining. HC *n* = 4, VEDOSS *n* = 10, SSc *n* = 6. (**B**) Quantification of CD45 staining, as outlined in Supplementary Fig. S5B, available at *Rheumatology* online. Mean ± s.e.m. (**C**) Correlation between semi-quantitative analysis of CD45 and MT staining for VEDOSS patients (Pearson correlation coefficients; R = 0.5881, *P* = 0.0738). (**D**) CXCL10 IHC staining for representative VEDOSS sample with low and high CD45 staining, along with HC and SSc sample (additional staining in Supplementary Fig. S6, available at *Rheumatology* online). HC *n* = 3, VEDOSS *n* = 10. Histological images: scale bar depicts 100 µm. VEDOSS: Very Early Diagnosis of Systemic Sclerosis; IHC: immunohistochemistry; MT: Masson’s trichrome

We have shown that increased ECM deposition is observed within VEDOSS skin prior to clinical detection. Skin biopsies from SSc typically show CD45+ perivascular infiltrate, which has been shown to correlate with early disease and progression of mRSS [32, 33]. VEDOSS biopsies (*n* = 10) showed a variable but overall higher level of CD45+ perivascular infiltrate in the dermis compared with HC (*n* = 4) (Fig. 3A, Supplementary Fig. S5A, available at *Rheumatology* online). Semiquantitative assessment of CD45 positivity showed comparable levels to the one observed in biopsies from patients classifiable as SSc according to the 2013 criteria (Fig. 3B, Supplementary Fig. S5B, available at *Rheumatology* online). Interestingly, semiquantitative assessment of CD45 infiltrate and MT staining in VEDOSS samples indicates a correlation between the two features (R = 0.588, *P* *=* 0.074) (Fig. 3C), suggesting that there is a possible pathogenic link between leukocytes infiltration and increased dermal collagen production (Fig. 3C).

We and others have documented signs of Type I IFN activation in skin biopsies from SSc patients and animal models of disease, which include increased expression of Type I IFN inducible proteins such as CXCL10, particularly apparent within the epidermis [34–39]. VEDOSS biopsies (*n* = 10) showed a variable expression of CXCL10, which paralleled the extent of CD45 infiltration (Fig. 3D, Supplementary Fig. S6, available at *Rheumatology* online), supporting the already published data indicating a chemotactic role of this protein in SSc. Indeed, 90% of VEDOSS samples show increased epidermis and dermal CXCL10 expression compared with HC (Supplementary Fig. S6, available at *Rheumatology* online). However, semiquantitative analysis of CXCL10 staining of dermis did not correlate with MT or CD45 staining in VEDOSS samples (data not shown).

### Serological analysis of VEDOSS patients show increased type I IFN activation and ECM remodelling

**Figure 4.**
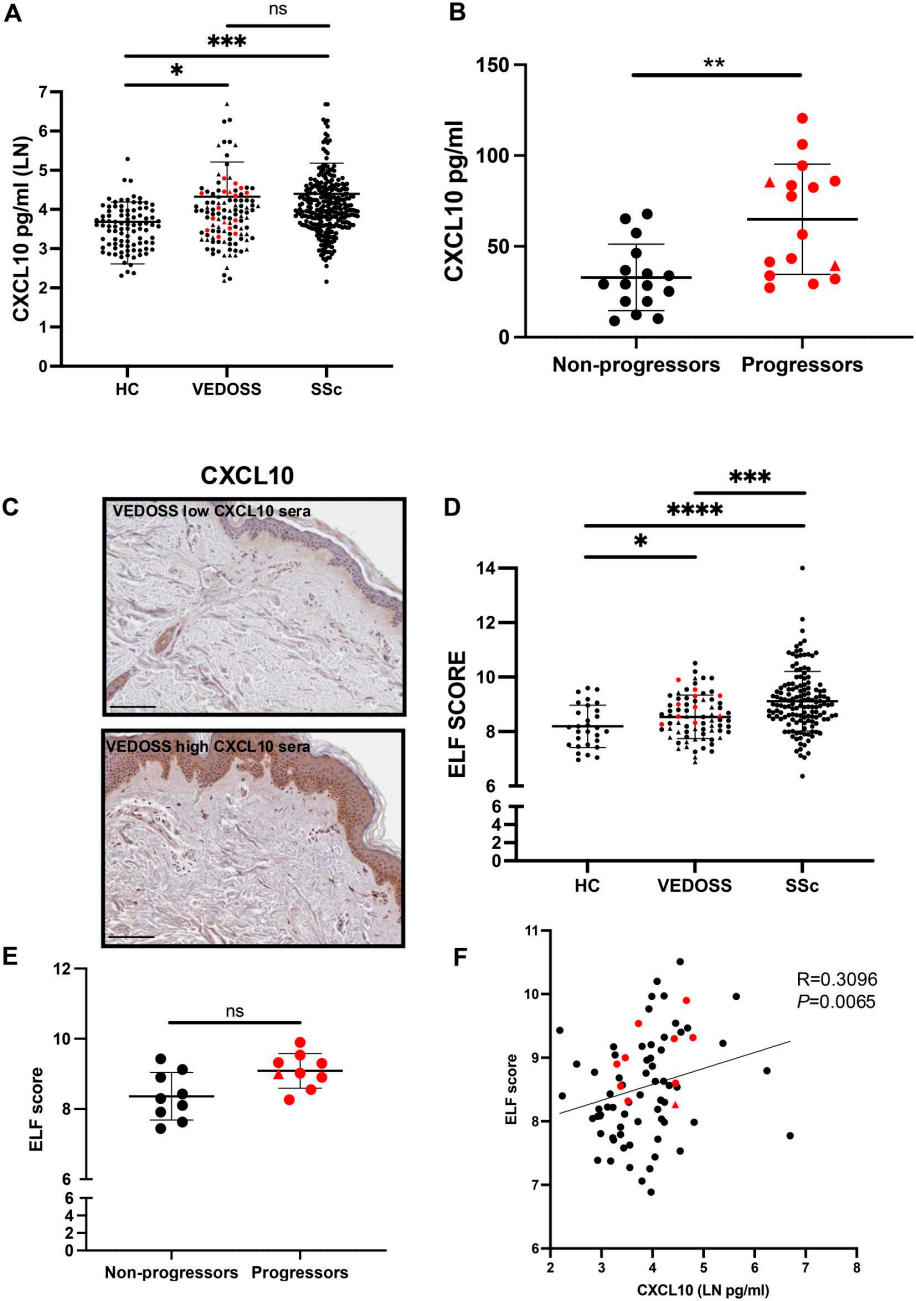
Serum of VEDOSS patients shows markers of increased Type I IFN activation and ECM remodelling, and CXCL10 linked to SSc progression. (**A**) CXCL10 sera levels in HC *n* = 93, VEDOSS *n* = 114 and SSc *n* = 284. Line and bars represent mean ± s.d. shown in natural log pg/ml. Red dots represent those individuals who progressed to SSc disease. Triangle dots highlight those with concurrent skin biopsy analysis. (**B**) Sera baseline CXCL10 levels in those that progressed to SSc disease, and in non-progressors (matched for age, gender and follow-up duration) (*n* = 16). (**C**) Representative CXCL10 IHC dermal staining of biopsies from VEDOSS patients with low and high CXCL10 sera concentration. All VEDOSS samples (and HC) are shown in Supplementary Fig. S6, available at *Rheumatology* online in order of increasing CXCL10 sera levels (as shown in A). (**D**) Available ELF score of those in (A) (combined PIIINP, TIMP1, HA) (HC *n* = 29, VEDOSS *n* = 76, SSc *n* = 143). (**E**) ELF score analysis in progressors verse matched non-progressors (matched for age, gender and follow-up duration) (*n* = 9). (**F**) Serological CXCL10 and ELF score Spearman correlation analysis in VEDOSS patients (*n* = 76). One-way ANOVA used for analysis and unpaired Student’s *t*-test for analysis (*P < 0.05, **P < 0.01, ***P < 0.001, ****P < 0.0001, ns: non-significant). VEDOSS: Very Early Diagnosis of Systemic Sclerosis; ECM: extracellular matrix; TIMP-1: tissue inhibitor of metalloproteinases 1; PIIINP: amino-terminal propeptide of type III procollagen; HA: hyaluronic acid

Type I IFN activation has been previously observed within the blood of VEDOSS patients (*n* = 19) [34]. Serological CXCL10, has been shown to be higher in VEDOSS compared with HC (*n* = 21) [15] and in another cohort (*n* = 34) when stratifying for active and late NVC changes and SSc progression within 5 years [16]. Building on our data on skin CXCL10 IHC and these published observations, we set out to extend this analysis in our population (*n* = 114, Table 1). VEDOSS sera showed mean ± s.d. 75.9 ± 107.8 pg/ml concentration of CXCL10 of comparable to SSc patients 85.07 ± 129.3 pg/ml (*n* = 284), and significantly higher than HC 39.90 ± 26.2 pg/ml (*P* *=* 0.01) (*n* = 93) (Fig. 4A). During median (IQR) 10.5 (21) months of follow-up, 16 patients (14%) progressed to fulfil SSc criteria. These patients showed a 2-fold higher concentration of CXCL10 compared with non-progressors matched by age, gender and follow-up period duration (64.9 *vs* 32.8 pg/ml, *P* *=* 0.0071) (Fig. 4B). Interestingly, within the limited number of biopsies available, high expression levels of CXCL10 paralleled high serum concentration of CXCL10 (*n* = 10) (Fig. 4C, Supplementary Fig. S6, available at *Rheumatology* online), indicating Type I IFN activation present in the dermis can be measured at the circulatory level.

It has recently been shown that serological markers of ECM remodelling are increased in VEDOSS (*n* = 42) [17]. We and others have previously shown that the protein biomarkers of ECM PIIINP, TIMP-1 and HA, along with the combined algorithm concentration known as the ELF score, are increased in SSc compared with HC and correlate with disease severity and fibrotic damage [40–42]. To determine whether there was any evidence of ECM turnover before disease manifestation, we analysed the available ELF score data from VEDOSS patients alongside HC and SSc (*n* = 76, 29 and 143, respectively). The mean ELF score in VEDOSS and SSc was significantly higher than in HC (8.54, 9.11 *vs* 8.19; *P* *<* 0.05, <0.0001, respectively), with VEDOSS values being significantly lower than SSc (*P* *=* 0.0002) (Fig. 4D). VEDOSS patients that progressed to SSc diagnosis within the follow-up period had an elevated, but not statistically significant, ELF score compared with non-progressors (Fig. 4E). Further, we observed a significant correlation between ELF score and CXCL10 serum concentration in VEDOSS (R = 0.3096, *P* *=* 0.0065) (Fig. 4F). Univariate analysis of clinical variables showed a significant (negative) correlation with DLCO% for both CXCL10 (R = –0.3580, *P* *=* 0.0005, *n* = 91) and ELF (R = –0.3541, *P* *=* 0.0033, *n* = 67) in VEDOSS patients (Supplementary Fig. S7, available at *Rheumatology* online).

### The presence of PF does not drive the biological SSc hallmarks in VEDOSS

**Figure 5.**
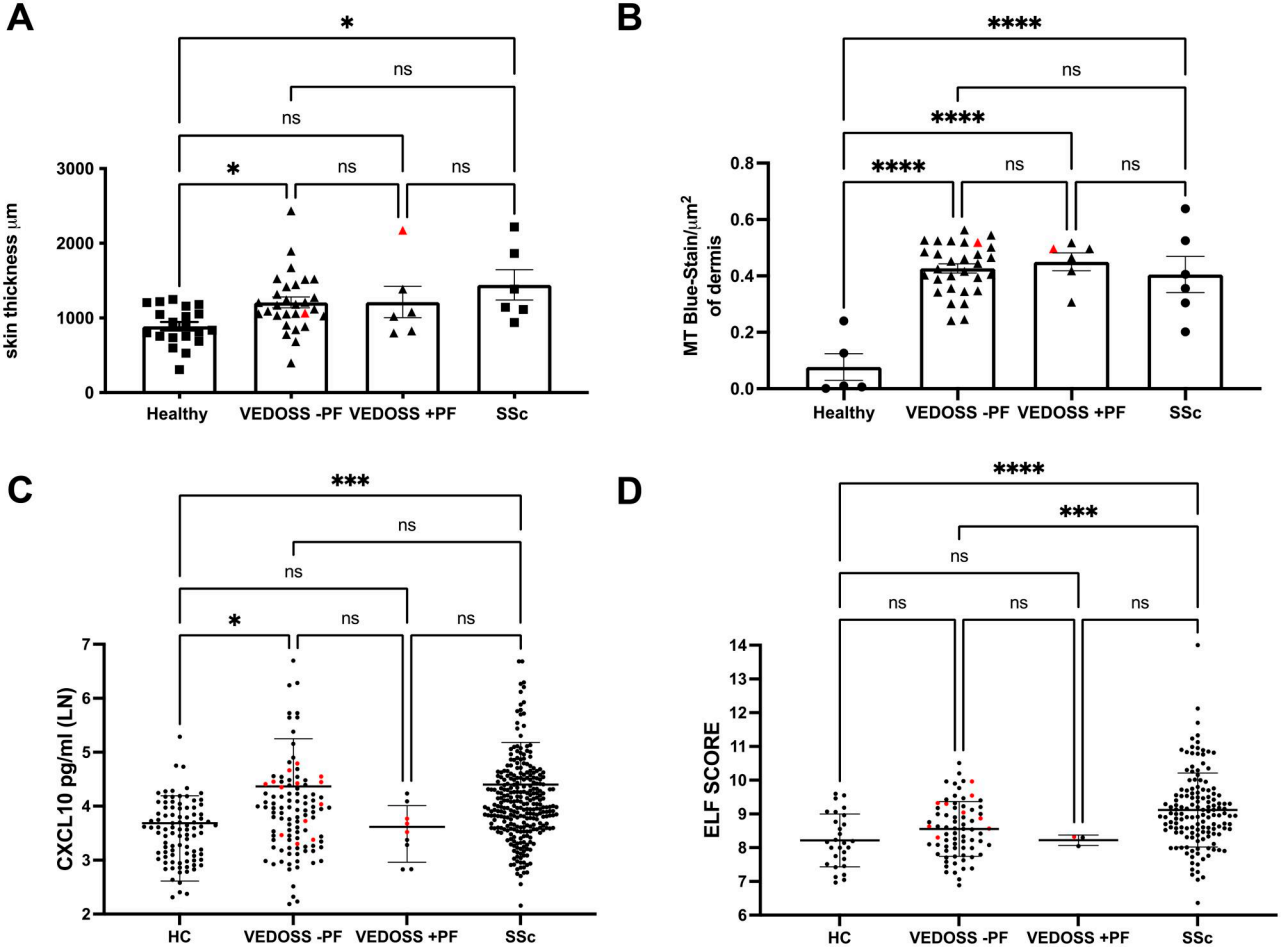
The presence of puffy fingers does not drive the biological SSc hallmarks in VEDOSS. (**A**) Skin thickness analysis of all suitable samples within the 3 categories with population (HC *n* = 20, VEDOSS *n* = 35, SSc *n* = 6), with VEDOSS sub-divided for presence (+PF) or absence (–PF) of puffy fingers. (**B**) Quantification of collagen staining by MT per µm^2^ of dermis plotted (HC *n* = 5, VEDOSS *n* = 36, SSc *n* = 6), with VEDOSS sub-divided for presence (+PF) or absence (–PF) of puffy fingers. (**C**) CXCL10 sera levels (natural log pg/ml) in HC *n* = 93, VEDOSS *n* = 114, and SSc *n* = 284, with VEDOSS sub-divided for presence (+PF) or absence (–PF) of puffy fingers. (**D**) Available ELF score of those in (C) (combined PIIINP, TIMP1, HA) (HC *n* = 29, VEDOSS *n* = 76, SSc *n* = 143), with VEDOSS sub-divided for presence (+PF) or absence (–PF) of puffy fingers. Graphs show mean ± s.e.m. (A, B) and mean ± s.d. (C, D), with single points represent individual biosamples, with red highlighting those baseline VEDOSS samples that progressed to SSc. One-way ANOVA used for analysis (*P < 0.05, **P < 0.01, ***P < 0.001, ****P < 0.0001, ns: non-significant). VEDOSS: Very Early Diagnosis of Systemic Sclerosis; HC: healthy controls; PF: puffy fingers; MT: Masson’s trichrome; ANOVA: analysis of variance; TIMP-1: tissue inhibitor of metalloproteinases 1; PIIINP: amino-terminal propeptide of type III procollagen; HA: hyaluronic acid

A larger proportion of VEDOSS patients in our biopsy group were associated with PF compared with the serum group, in addition PF have been associated with an increased risk of progression to SSc [14], thus we wanted to assess whether PF were a driving factor of the biological SSc hallmarks in our analysis of VEDOSS. There was no significant difference between the skin thickness and ECM deposition between VEDOSS with and without PF, and VEDOSS with no PF maintained this increased dermal collagen compared with HC (Fig. 5A, B). Samples analysed for CD45 and CXCL10 did not comprise of samples from PF positive patients, thus is not dependent on the presence of this clinical feature.

Performing CXCL10 serological analysis between VEDOSS PF+ and PF– subgroups showed no significant difference, and VEDOSS PF– maintained increased CXCL10 compared with HC (Fig. 5C). VEDOSS PF+ lost the significant difference to HC (Fig. 5C). For ELF score analysis, there is no significant difference between VEDOSS PF+ and PF–, however the mean differs 8.22 compared with 8.55, respectively (Fig. 5D). The significant increase to HC is lost in both VEDOSS subgroups (Fig. 5D).

## Discussion

In this study we show that VEDOSS patients already show biological signs of SSc, supporting a biological diagnosis of SSc. We show here that despite no clinically detectable skin thickening, biopsies from VEDOSS patients show increased collagen fibers and increased dermal thickness, and a pattern of increased perivascular infiltrate. This observation supports the notion that the natural history of SSc extends before the time patients fulfil classification criteria and the biological processes leading to skin involvement are already active at the VEDOSS stage. This observation is also consistent with published data showing an SSc gene signature in clinically not affected skin and changes in optical coherence tomography features of the skin in patients with clinically undetectable increase of skin thickening (mRSS = 0) [37, 43].

Previous serological analysis of CXCL10 and ECM remodelling has been shown to be dysregulated in VEDOSS in smaller cohorts [15–17], however in this study we have combined autologous sera and dermal analysis to gain further insight into the initiation of SSc progression. We show for the first time that the VEDOSS dermis has a similar level to SSc of increased CD45+ infiltration compared with healthy tissue, which shows a trend matched to collagen levels, suggesting that at this preclinical stage of SSc, fibrosis and inflammation co-occur. However, a greater sample size is required to confirm this analysis. We have previously shown that human CD45+ cells, specifically plasmacytoid dendritic cells, directly contribute to CXCL10 expression and skin fibrosis [39]. In this sense, we could speculate that the CD45+ infiltrate directly contributes to the increased ECM deposition in VEDOSS. The significant correlation between CXCL10 and ELF score levels in the sera of these patients also supports this notion. We also show that the clinical feature of PF is not a driver of these biological hallmarks in the studied VEDOSS biosamples, in terms of increased ECM deposition and serological CXCL10 levels. Nevertheless, it would be interesting to determine from connectome analysis of skin RNAseq or from spatial transcriptomics, which are the cells that interact directly with CXCL10 and address whether the increased expression is linked to profibrotic signalling, perivascular infiltration and/or impaired angiogenesis. Notably, while our studies confirm the value of CXCL10 in the VEDOSS population, this is not a specific marker for SSc as increased levels of CXCL10 have been shown in SLE, DM as well as localized scleroderma [44–46].

We found it particularly interesting that dermal fibroblasts isolated from VEDOSS biopsies already showed the typical profibrotic activation that has allowed to dissect the molecular mechanisms of fibrosis in SSc [28]. This observation, together with the ECM assessment and ELF score analysis, supports the notion that profibrotic activity is present in VEDOSS patients before fibrosis is clinically detectable at this stage. It is interesting to note that we detected increased skin thickness despite patients having mRSS = 0. While this is in line with several features detected by RNA in clinically not-affected skin [37], it also supports the notion that the mRSS may have a high threshold for skin thickness detection and more sensitive tools are needed for early detection. We did observe increase serological ELF score in VEDOSS, however, besides ELF, a number of biomarkers have been linked to fibrogenesis, such as CTGF. Studies are on-going in our unit to identify early markers of fibrogenesis in the VEDOSS population and their predictive role on clinical progression.

This study also extends our previous analysis of serum CXCL10 during SSc progression [16]. The increased CXCL10 concentration in VEDOSS patients who progressed *vs* patients that did not progress to SSc during our observational study supports the notion that a higher Type I IFN or innate immunity drive may be linked to progression to clinically detectable signs. However, it is important to note that patients not meeting SSc classification today still have the potential to progress within 5 years [14]. Thus, larger and longer studies are needed to build predictive models that enrich the current predictive value of VEDOSS clinical signs, as well as assessment with other established clinical markers.

The very simple observations of this study, while supporting the concept of biologically active disease at the VEDOSS stage, raise several questions that deserve further research. Is there a specific signature detectable in the skin that changes at the time of clinical progression? Is the lack of progression to clinically detectable skin involvement an active process or simply the effect of a milder pathology? The observations of this study have informed an extended longitudinal multiomics study both on VEDOSS sera and skin biopsies that is currently ongoing and will help in addressing these questions.

In conclusion, this pilot study on VEDOSS biosamples clearly shows early detection of biological hallmarks of SSc, offering a biological validation to the clinical observations of the VEDOSS study and supporting further research to exploit this window of opportunity for delaying the onset of clinical signs of SSc. Our data support the growing recognition of the preclinical phase of SSc [47] and support the identification of early biomarkers that could aid prediction of imminent progression and be used for enriching strategies in clinical intervention.

## Supplementary Material

keae698_Supplementary_Data

## Data Availability

Data are available upon reasonable request to the corresponding author. All data relevant to the study are included in the article or uploaded as supplementary information.
