# Supplementary material for: Biological hallmarks of systemic sclerosis are present in the skin and serum of patients with Very Early Diagnosis of Systemic Sclerosis (VEDOSS)
Source: Rheumatology (Oxford). 2024 Dec 19;64(6):3606–17. doi: 10.1093/rheumatology/keae698 (PMC12107078; doi:10.1093/rheumatology/keae698)
Supplement: keae698_Supplementary_Data [file keae698_supplementary_data.pdf]

A

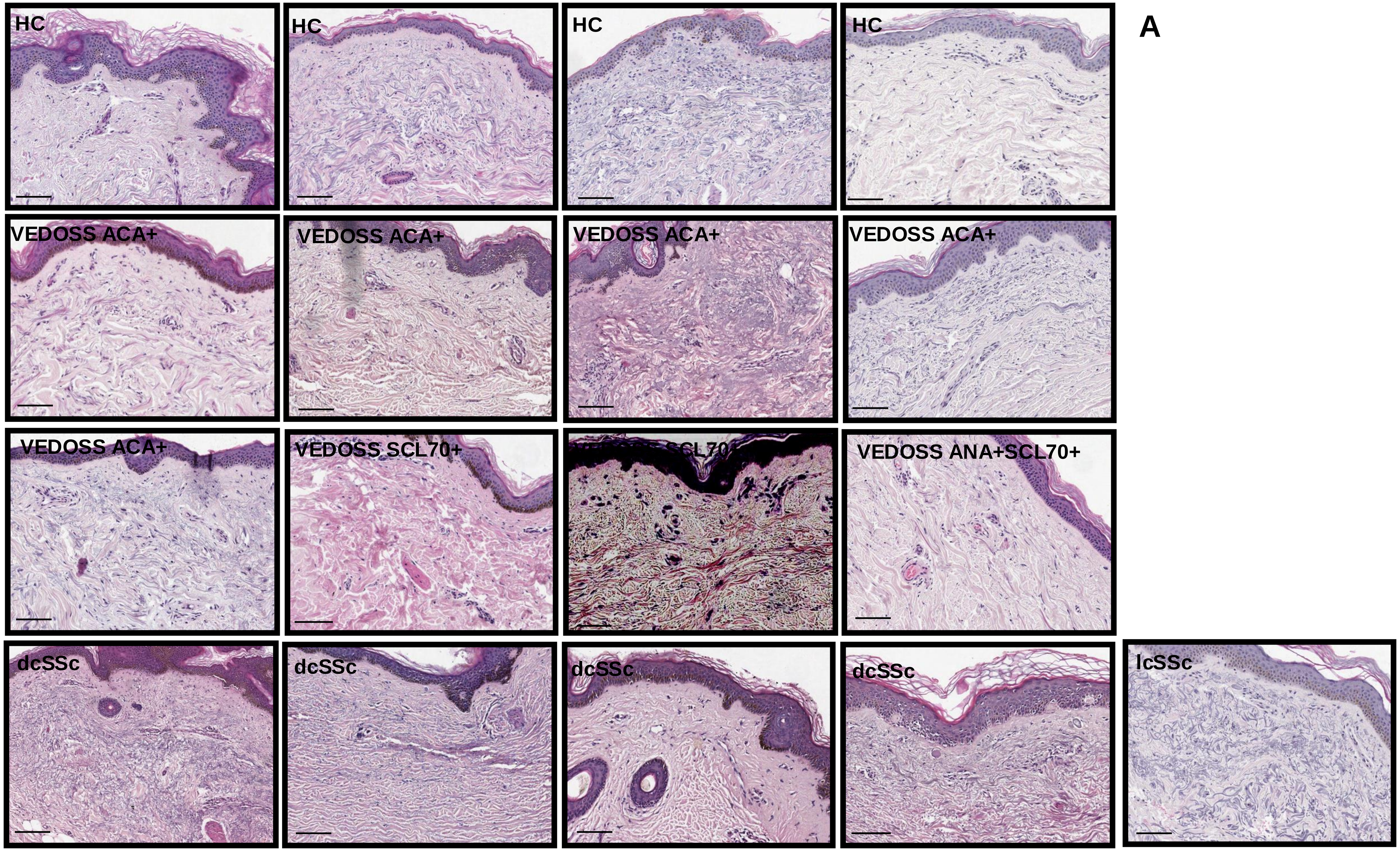

Histological feature analysis on H&E sections	HC (n=20)	VEDOSS (n=35)
Normal	80% (16)	43% (15)
SSc features identified	20% (4)	57% (20)
Collagen changes	10% (2)	40% (14)
Loss of fat around eccrine coils	15% (3)	20% (7)
Increased cellularity	5% (1)	17% (6)
Increased perivascular inflammation	5% (1)	14% (5)
SSc diagnosis evident	0% (0)	9% (3)

B

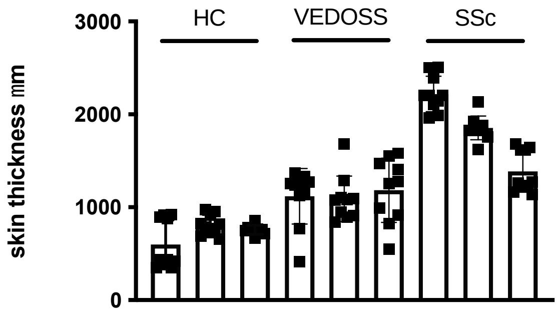

Supplementary Figure S1 Haematoxylin and eosin (H&E) histology based assessment of SSc features in VEDOSS skin. (A) H&E staining of VEDOSS (n=10) and healthy control (n=5) skin samples, along with 6 SSc samples to compliment Fig 1A. Scale bar depicts 100 µm. Table illustrates the blinded pathological analysis of H&E staining of the skin biopsy cohort (suitable specimens selected based on samples covering the full morphology of the skin). Normal classification based on features of waist and collagen structures and no features of SSc observed; such as loss of fat around eccrine coils, increased dermal cellularity, increased perivascular inflammation, and dense/altered collagen patterns. Of those not classified as normal, the features of SSc have been broken down to previously mentioned observations. Observations are positive if mild-severe observed. **(B)** Skin thickness mean and standard deviation of 3 representative skin biopsy samples for each category, HC, VEDOSS and SSc. Single dots represent individual measurements within a single biopsy.

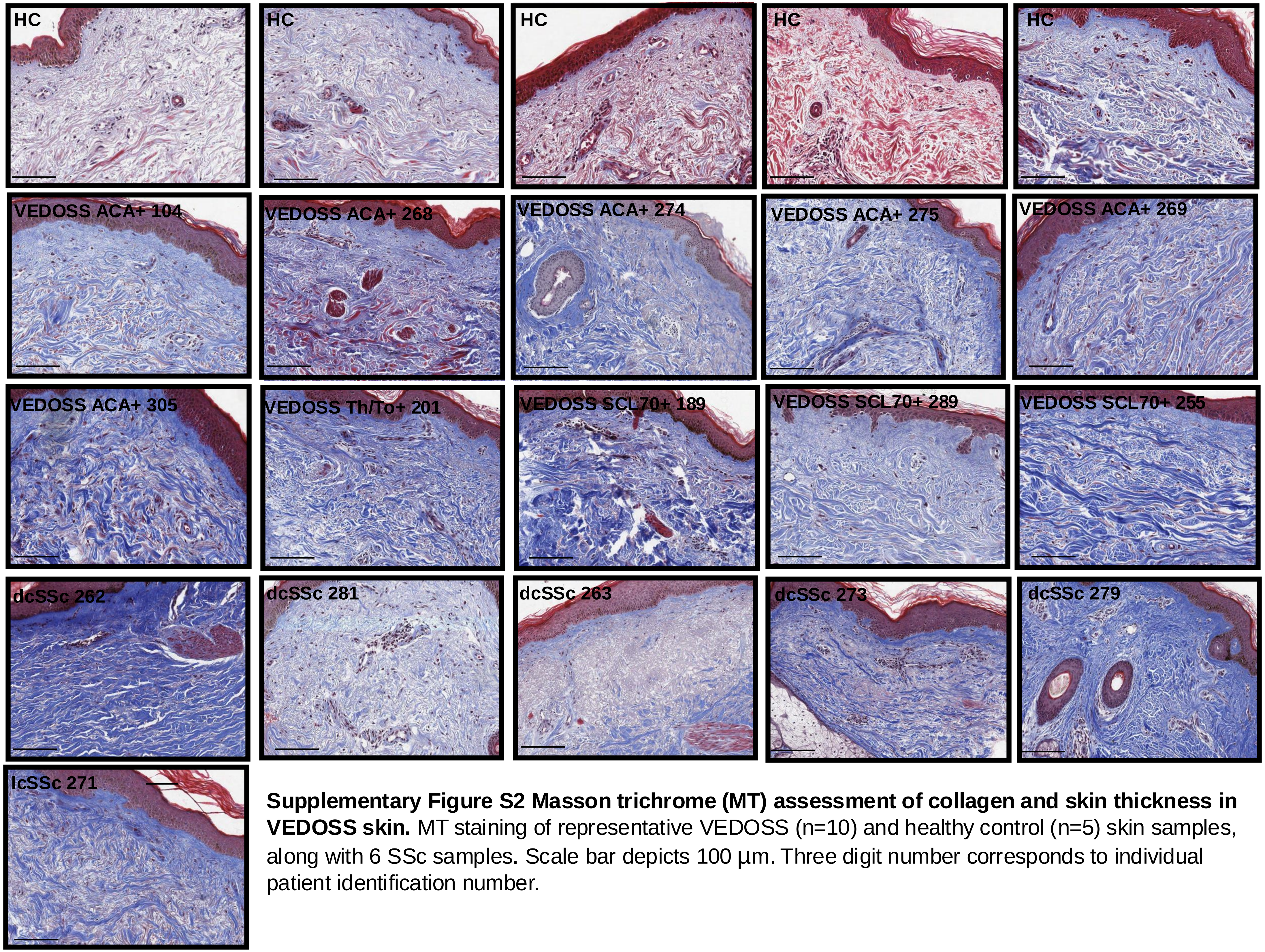

Supplementary Figure S2 Masson trichrome (MT) assessment of collagen and skin thickness in VEDOSS skin. MT staining of representative VEDOSS (n=10) and healthy control (n=5) skin samples, along with 6 SSc samples. Scale bar depicts 100 µm. Three digit number corresponds to individual patient identification number.

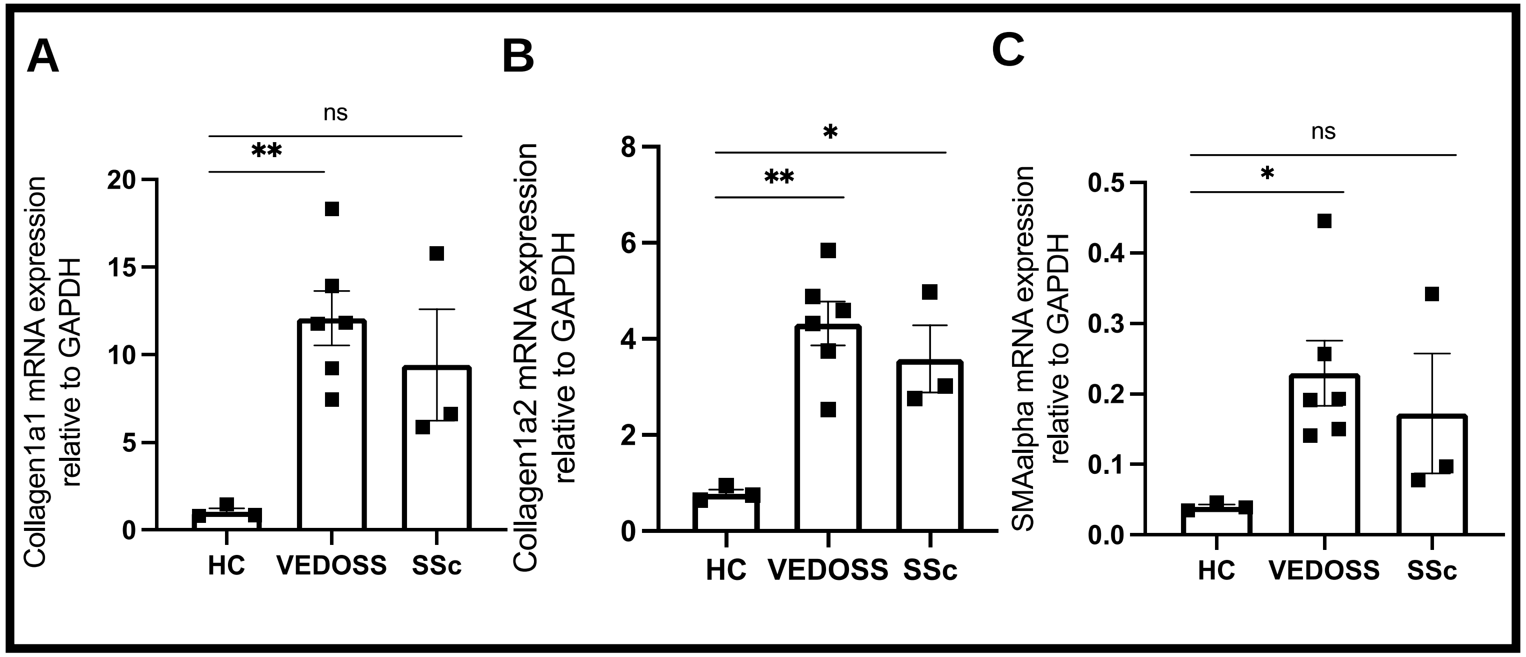

Supplementary Figure S3. Immortalised explanted dermal fibroblasts from VEDOSS patients maintain increased collagen and SMA compared to Healthy control. 3mm skin punch biopsies were taken from the forearms of 6 VEDOSS patients and 3 healthy controls (HC) and 3 SSc patients (dcSSc) and dermal primary fibroblasts explanted. Cells were immortalized with retroviral expression of human telomerase (HTERT). (A-C) Collagen 1a1, collagen 1a2, and SMAalpha mRNA expression in starved media relative to GAPDH housekeeping gene. Data shows biological replicates of each subset. Data shows mean of subsets +/- SEM. Unpaired student t test used for analysis (ns = non-significant; P<0.05 = *; P<0.01 = **).

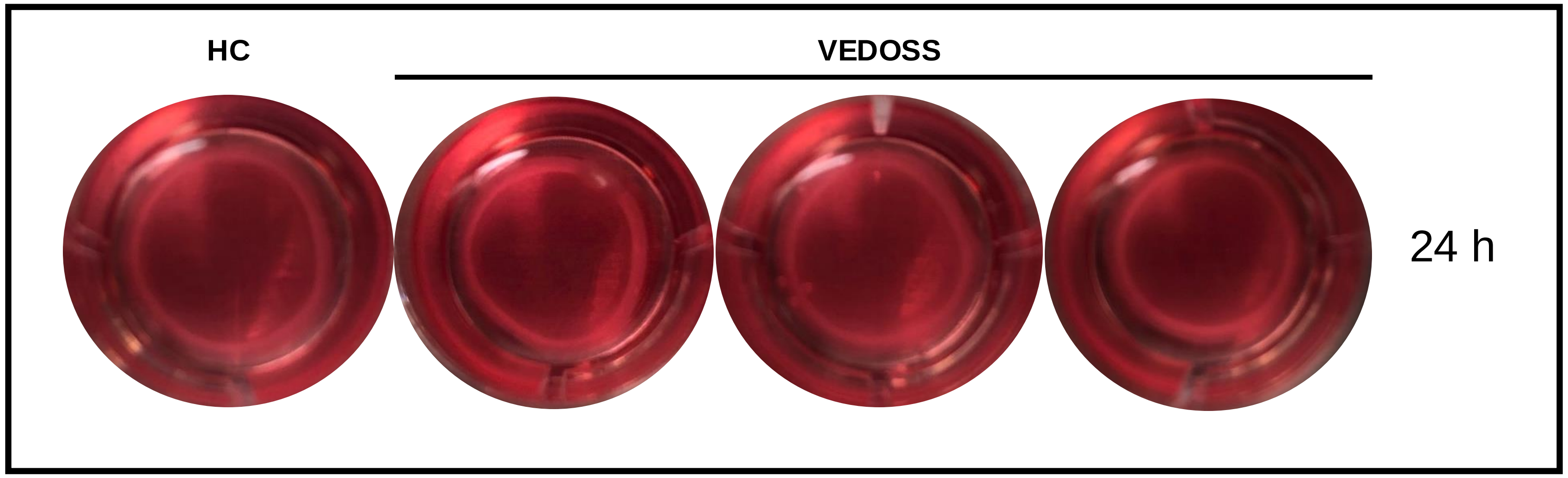

Supplementary Figure S4. Gel contraction images. Example images of HC (n=1) and VEDOSS (n=3) samples at 24 h for Figure 2E.

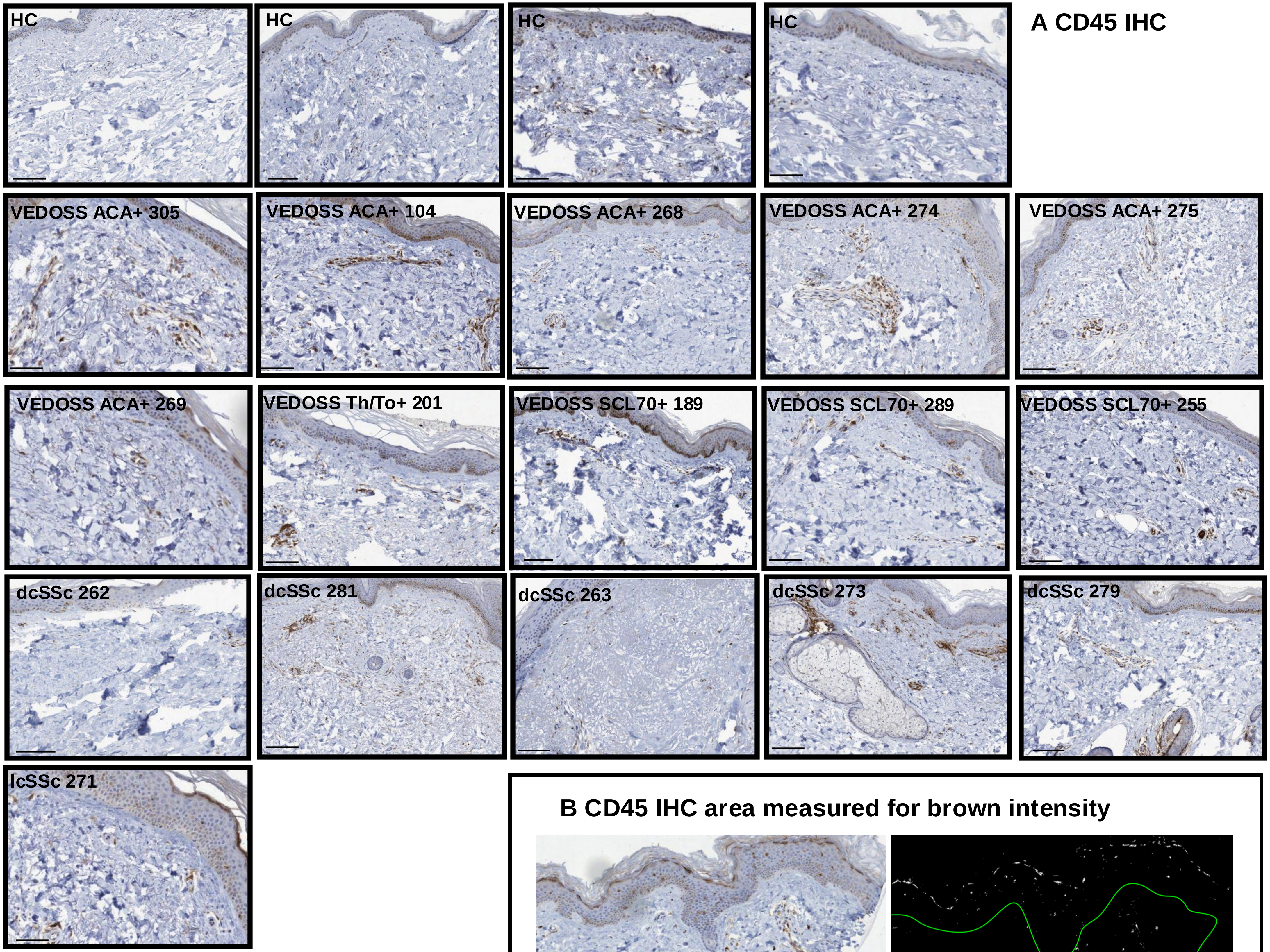

B CD45 IHC area measured for brown intensity

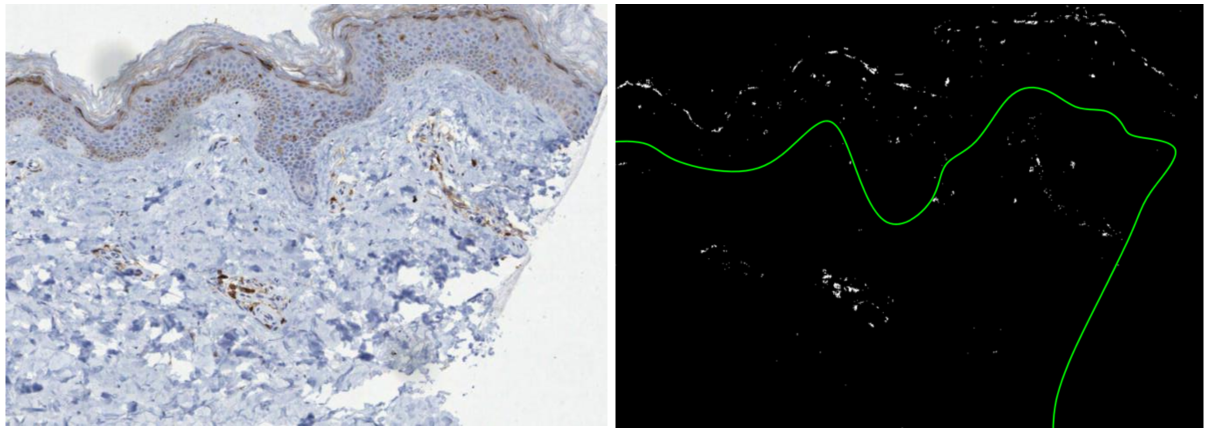

Supplementary Figure S5. Perivascular infiltration analysis with CD45 IHC of skin.
(A) CD45 IHC staining of VEDOSS (n=10) skin samples along with HC (n=5) and SSc (n=6). Scale bar depicts 100 μm. Three digit number corresponds to individual patient identification number. (B) computer analysis of levels; Excluding epidermis and sites on hemorrhage.

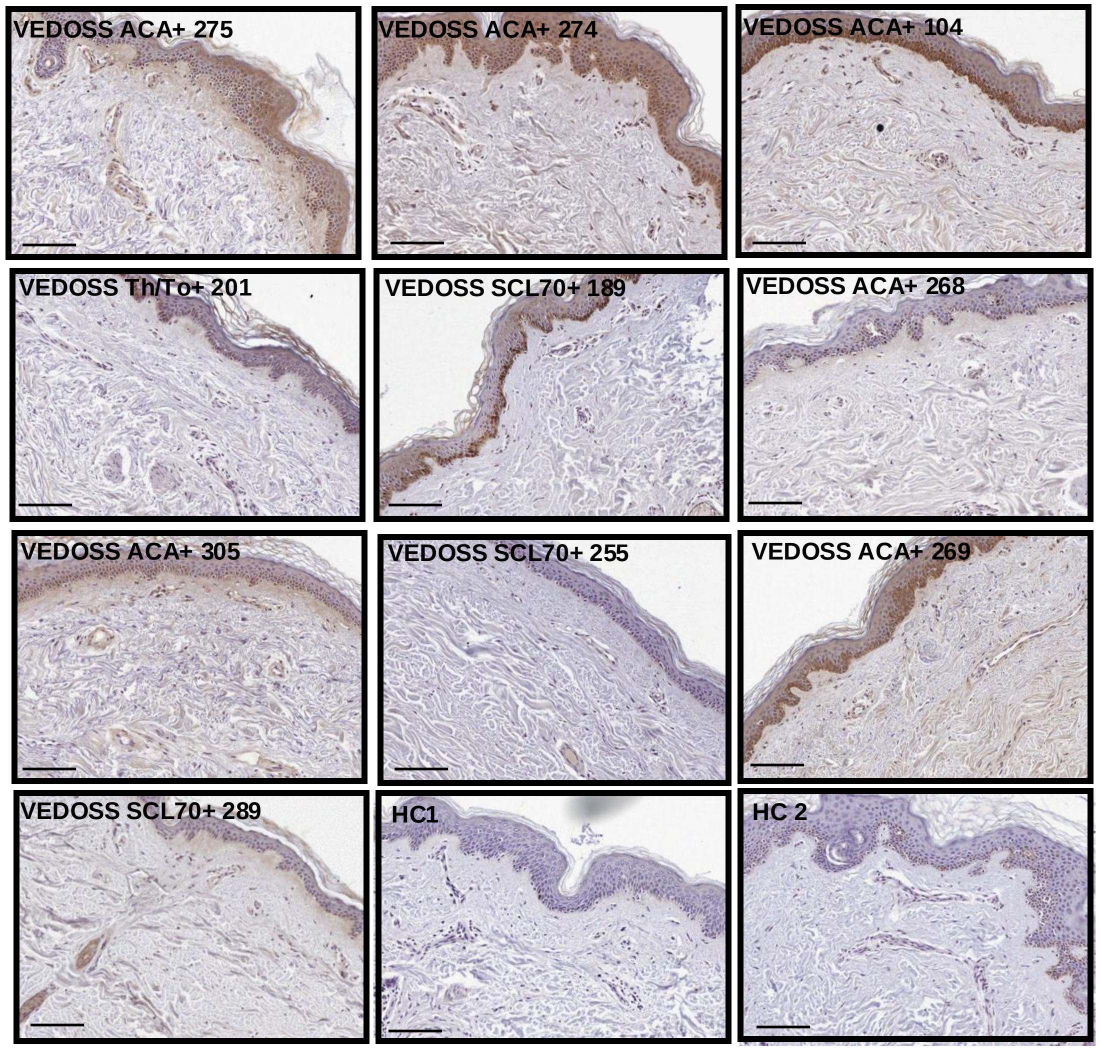

Supplementary Figure S6 CXCL10 IHC and sera correlation. CXCL10 IHC staining of VEDOSS samples (n=10) in order left to right for decreasing CXCL10 sera levels (Figure 4B), and 2 representative HC biosamples. Scale bar is 100 µm. Three digit number corresponds to individual patient identification number.

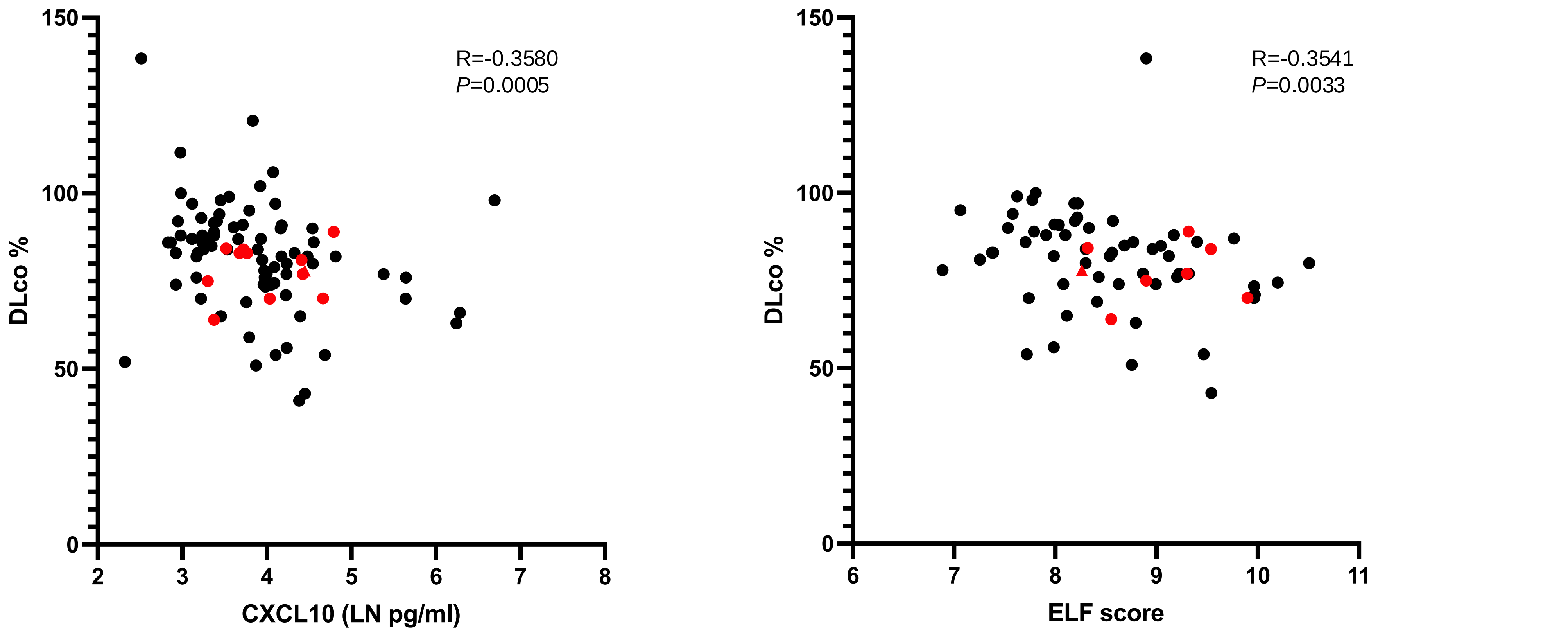

Supplementary Figure 7 CXCL10 and ELF score serological correlation to Dlco% in VEDOSS. Dlco data of available VEDOSS patients (n=91) was correlated to serological CXCL10 levels (LN = natural logarithm of pg/ml) and to ELF score (n=67). Spearman correlation test performed.
